# Treatment of Iris Hypoplasia Using Semiconductor Diode Laser in the Horse Under Standing Sedation

**DOI:** 10.1155/crve/4021047

**Published:** 2025-03-24

**Authors:** Ethan M. Hefner, Riccardo Stoppini, Richard J. McMullen

**Affiliations:** ^1^JT Vaughan Large Animal Teaching Hospital, College of Veterinary Medicine, Auburn University College of Veterinary Medicine, Auburn, Alabama, USA; ^2^Animal Eye Care of the Lowcountry, Ladson, South Carolina, USA; ^3^Equine Ophthalmology Consultant, Cascina Gufa Equine Clinic, Merlino, Lodi, Italy; ^4^Equine Department, University of Zurich, Zurich, Switzerland

## Abstract

**Objective:** This study is aimed at describing two unique case presentations of iris hypoplasia and the use of semiconductor diode laser for treatment in the horse.

**Animals Studied:** One 20-year-old American Quarter Horse gelding and one 11-year-old pony mare were studied.

**Results:** The presenting clinical signs, surgical technique, and postoperative results for two cases of iris hypoplasia treated with diode laser are described. Immediate postoperative complications—postoperative ocular hypertension (POH), corneal edema, and epithelial bullae—were possible anticipated effects but were controlled with topical medications in the first case. Following treatment, endothelial contact of the iris was eliminated, and a functional pupil was established in the first case. Anterior synechia occurred long-term but did not lead to pupil obstruction. Left untreated, the iris hypoplasia in the right eye progressed to a degree like that seen upon initial presentation in the left eye.

In the second case, the area of the distended, hypoplastic iris was reduced, and the pupil remained functional.

**Conclusions:** Diode laser ablation of hypoplastic iridal tissue was effective at eliminating anterior synechia in both cases presented here. The better functional results in Case 2 suggest that early intervention may result in more favorable outcomes.

## 1. Introduction

Iris hypoplasia is often described as an incidental finding in the dorsal iris of horses with heterochromic (blue) irides, although they may less frequently be present ventrally and in eyes with brown irides. They manifest as upraised darker areas of the affected iris representing thinning of the iris stroma. Iris hypoplasia is confirmed using retroillumination and observation of the tapetal reflection through the thin and/or fenestrated iris stroma. Ocular ultrasonography can be confirmatory in cases where retroillumination is ambiguous [1–3].

While iris hypoplasia is reported as a nonprogressive condition that is not associated per se with pain or vision impairment, this disease can clearly have a spectrum of presentations between affected horses and even between eyes from the same animal, as seen in Case 1 of the present report [1]. Historical treatment has been removed of the cyst wall if any treatment was performed [2]. Anecdotally, pharmacological dilation of the pupil may improve aqueous outflow but may cause photophobia and impair vision in bright lighting conditions. Marked iris atrophy may impact vision due to the increased resistance of aqueous humor flow when the pupil is miotic, resulting in the iris stroma being pushed anteriorly by the aqueous humor. Histologically, these appear as a thin area of the iris with poorly developed iris dilator muscle (often resulting in a miotic pupil), with poorly pigmented iridal tissue [3].

The two cases presented in this case report represent a unique form of hypoplasia, which is more typical of the problems associated with anterior uveal cysts in which the iridal tissue enlarges so much it begins to obstruct vision (sometimes causing behavioral change–head shaking) or causes intraocular damage (endothelial contact). Uveal cysts causing clinical problems are routinely treated with neodymium–yttrium aluminum garnet (Nd:YAG) laser or diode laser deflation [4–6]. While the suspected pathogenesis of aqueous buildup in the two circumstances is different, the goals for therapy were to recreate a functional pupil, redirect aqueous flow, and minimize/eliminate the consequences of unwanted contact between the iridal tissue and the corneal endothelium. We hypothesized that diode laser ablation of the atrophic iridal tissue following viscoelastic dissection of the iris stroma from the corneal endothelium (Case 1) would effectively create flow holes to allow aqueous to flow from the posterior chamber into the anterior chamber, thus resulting in a return of the dorsal iris stroma to a more natural position.

## 2. Case Report

### 2.1. Case 1

A 20-year-old Quarter Horse gelding presented to the Auburn Large Animal Teaching Hospital for structural changes to the iris of his left and right eyes (ODs) and a 2-month history of decreased vision in the left eye (OS). While the horse seemed visual, the owners noted that when barrel racing, he had recently begun swinging wider than normal (particularly to the left), which the owner believed was related to decreased vision in the OS.

On ophthalmic examination, the following abnormalities were noted: absent direct and consensual pupillary light reflex (PLR) in the OS and dorsal superficial corneal neovascularization in both eyes (OU). In the OD, there was perilimbal endothelial fibrosis at 1 o'clock and midstromal axial fibrosis. Mild iris hypoplasia was noted OD.

**Figure 1:**
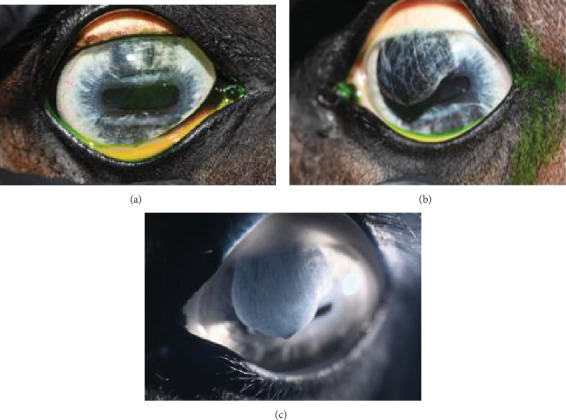
Digital color images (a) OD and (b) OS. Note mild superficial corneal vascularization and dorsal iris hypoplasia OD compared with severe iris hypoplasia, anterior synechia, and limited functional pupil with overhanging iris OS. (c) Digital infrared image OS of the hypoplastic iris. Note the translucent dorsal iris tissue.

**Figure 2:**
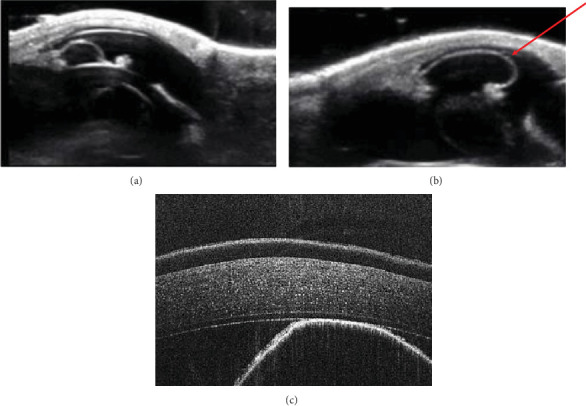
Ocular ultrasonography (a) OD and (b) OS. (c) Preoperative OCT OS. Note anterior displacement of iris towards endothelium dorsally (cyst-like region in the left side of image). The iris hypoplasia is (a) mild OD and (b) severe OS with iridal tissue approximating the corneal endothelium. Red arrow indicates the termination of the anterior synechia. Ocular ultrasonography was useful in determining the proximity of the atrophied iridal tissue to the cornea (decrease in anterior chamber depth). Additionally, distension of the posterior chamber, directly below the surface of the atrophied iris and perinuclear cataracts, could be readily identified (not shown). (c) Optical coherence tomography OS shows the hyperreflective surface of the hypoplastic iris contacting the corneal endothelium, in direct contact with Descemet's membrane, towards the bottom right of the image.

The anterior chamber OS was shallow with iris hypoplasia resulting in direct contact with the dorsal third of the corneal endothelium. Ventrally, vitreal presentation into the anterior chamber containing suspended pigment and an incipient perinuclear cataract was also noted. Color and infrared photographs were obtained OU (Figure 1). Ultrasound images were obtained with a 10-mHz (Canon Aplio i18LX5, Tustin, California) linear probe using a transpalpebral approach as well as with a standoff using a gloved finger distended with distilled water. Optical coherence tomography (EnvisuC2300; Bioptogen, Morrisville, North Carolina) was also performed to evaluate the cornea (Figure 2).

#### 2.1.1. Procedure: Anterior Synechiolysis Using Viscoelastic Dissection and Diode Laser Ablation

The horse was sedated with 0.01 mg/kg detomidine, 0.01 mg/kg butorphanol intravenously, and 0.027 mg/kg butorphanol intramuscularly. He received 1.1 mg/kg flunixin meglumine IV preoperatively. Auriculopalpebral and frontal supraorbital local eyelid and retrobulbar blocks were performed using 1 mL, 0.5 mL, and 10 mL carbocaine (2%), respectively. An eyelid retractor was placed following routine sterile preparation (dilute baby shampoo, 1% betadine solution, and sterile saline rinse). A 30-gauge needle/1 mL syringe combination was used to inject 2.0 mL viscoelastic (an-bfh 2.2% sodium hyaluronate, Albomed GmbH, Schwarzenbruck, Germany) between the hypoplastic iris and cornea. The wave of viscoelastic was used to separate the anterior synechia at the 12 and 1 o'clock positions. A 21-gauge needle was inserted through the limbus at the 12 o'clock position to create an opening for a nucleus rotator (Y-horizontal large, Anvision, Salt Lake City, Utah). The nucleus rotator was advanced past and across the ventral-most area of anterior synechia and swept ventrally and temporally to separate the iris and corneal endothelium.

**Figure 3:**
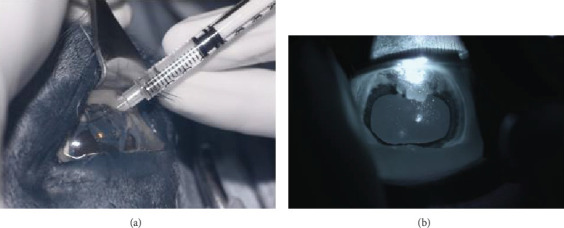
(a, b) Intraoperative digital infrared images OS. (a) Intracameral viscoelastic wave is created using a 30-G/1-mL insulin syringe combination to perform synechiolysis OS. (b) Diode laser focused on the dorsal hypoplastic iris (810 nm diode laser energy is visible using near-infrared digital imaging (Nikon Z6, full-spectrum conversion (http://LifePixel.com) and external standard (720 nm) IR filter (http://LifePixel.com)) showing constriction and coagulation of target tissue. Note the degree of mydriasis achieved. Pupil function was also returned to within normal limits following the procedure.

Following synechiolysis, infrared diode laser deflation and coagulation of the dorsal hypoplastic iris with anterior synechia and cyst-like presentation (Figure 3) was irradiated with infrared laser energy using a FOX A.R.C. diode laser emitting light with a wavelength of 810-nm using a laser indirect ophthalmoscope (LIO) and + 20D condensing lens. Power output was maintained at 1.5 W, in 1.5 s cycles (21 cycles total) until a total of 47.8 J was delivered. The power was increased to 2.0 W and 1.5 s cycles (118 cycles total) until a total of 402 J were delivered. Contraction and coagulation of the tissue were observed at the higher energy. No holes were visible in the iris tissue during the procedure.

Intraoperative complications included subconjunctival hemorrhage and mild chemosis. Preoperative intraocular pressure (IOP) (Tonovet, Icare, Vanna, Finland) was 16 mmHg, and immediate postoperative IOP was 9 mmHg. The horse was returned to his stall and received neomycin, polymixin B, and bacitracin hourly until he began blinking again and every 8 h overnight. He was transitioned to oral flunixin meglumine 1.1 mg/kg by mouth every 12 h.

**Figure 4:**
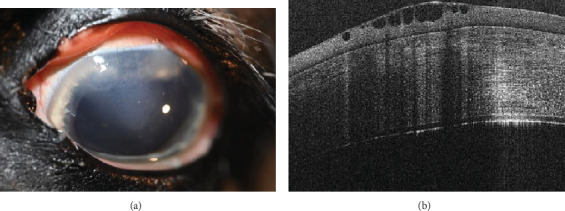
(a, b) Twenty-four hours postoperative digital color image and OCT of OS. (a) Axial edema with multifocal superficial bullae and perilimbal superficial vascularization. Persistent mydriasis. (b) Optical coherence tomography showing a cluster of hyporeflective bullae within the corneal epithelium and shadowing the posterior corneal stroma.

Corneal edema centered around the dorsonasal cornea with superficial, epithelial bullae was noted on ophthalmic examination and during OCT the following morning (Figure 4). The anterior chamber contained a mixture of viscoelastic and fibrin, and the IOP was 51 mmHg. The horse was returned to his stall and received 0.1 mL prednisolone acetate 1% hourly in the OS for the next 4 h. His IOP decreased during re-evaluation 4 h, postoperatively, but remained elevated at 40 mmHg. Prednisolone acetate was continued hourly and 0.1 mL dorzolamide/timolol was instilled OD hourly for the following 5 h, at which point his IOP had decreased to 31 mmHg. The following morning, the IOP was 30 mmHg, the corneal bullae had resolved, and the corneal edema was less prominent OS. The fibrin was gone, and there did not appear to be any viscoelasticity remaining in the anterior chamber. The horse was discharged on a tapering course of flunixin–meglumine over the next 10 days, neomycin, polymixin B, and bacitracin three times daily for 5 days, and topical prednisolone acetate OS twice daily for 5 days and then once daily for 5 days.

**Figure 5:**
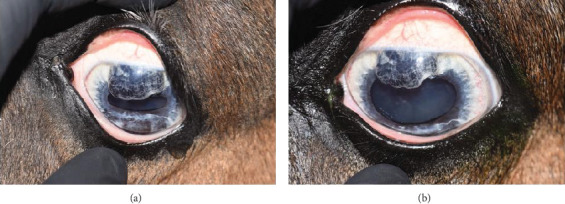
(a, b) Digital color images. (a) OS 50 days postoperatively prior to pharmacological mydriasis. Note that the corneal edema and bullae have completely resolved. The overhanging atrophic iris has been significantly reduced in size. (b) OS following pharmacological mydriasis with 1% tropicamide ophthalmic solution. Some redundant tissue remains in approximation with the cornea dorsally, but mydriasis permits a more thorough evaluation of the lens and fundus.

**Figure 6:**
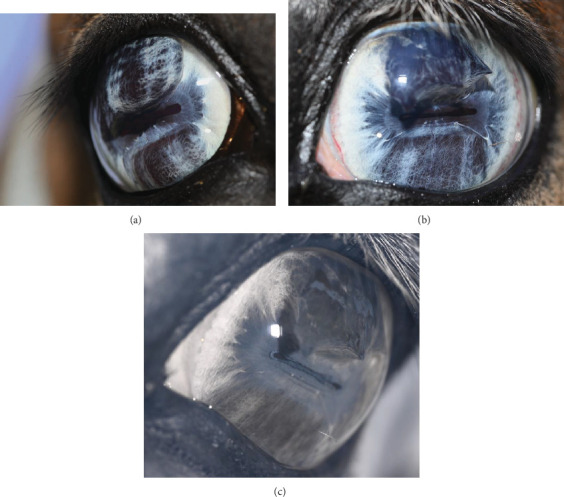
Digital color images (a) OD, (b) OS, and (c) infrared OS. (a) Digital color images OD showing the progression of iris hypoplasia in dorsal and ventral iris stroma, resulting in intense miosis. (b) Digital color image OS 995 days postoperative. Posterior synechia dorsally, not distorting the pupil margin or overhanging the pupil. (c) Infrared image OS demonstrating how thin the dorsal and ventral iris tissue is. No apparent ballooning forward of the ventral iris.

A recheck examination was performed 50 days after surgery. The dorsal iris had again ballooned anteriorly and was closely approximated with the endothelium, but the pupil margin was more mobile, allowing for a positive PLR and more complete mydriasis, facilitating a more complete examination of the lens and fundus prior to pharmacological dilation. Following the topical application of 1% tropicamide, the pupil became moderately (2+) mydriatic and no longer had the most ventral portion of anterior synechia (Figure 5). The patient was discharged with instructions to use tropicamide once daily in the evening to facilitate mydriasis and reduce the risk of further synechia formation. The patient presented 995 days after the initial presentation for a recheck examination. At this time, the OD had developed a cystic presentation of iris hypoplasia of both the dorsal and ventral iris, causing significant miosis (Figure 6). The left eye had anterior synechia and miosis, with mild corneal edema nasally. However, there was no redundant overhang of the iridal tissue across the central pupil, and no progression of lens instability was noted. The horse did not show any signs of ocular discomfort or behavioral changes. He had retired from barrel racing, and the owners declined any additional intervention.

### 2.2. Case 2

**Figure 7:**
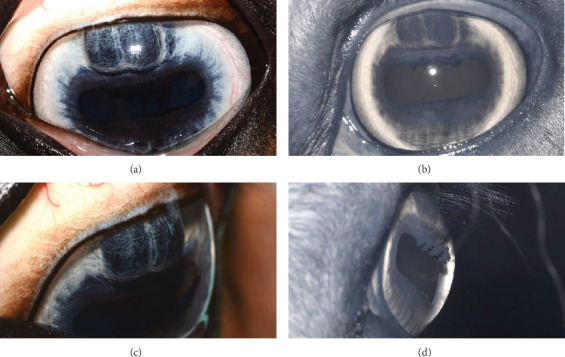
Case 2 (a) frontal color and (b) infrared images. (c) Oblique color and (d) infrared preoperative images. Dorsal iris hypoplasia is visible in the left eye without corneal endothelial contact.

**Figure 8:**
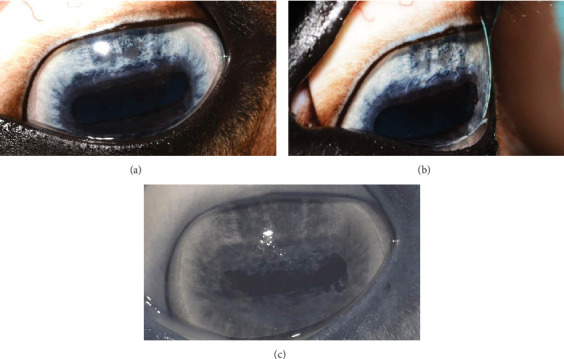
(a, b) Digital and (c) infrared images of Case 2 after laser photoablation. The iris bullae (iris hypoplasia) are completely flattened with normal iris color.

**Figure 9:**
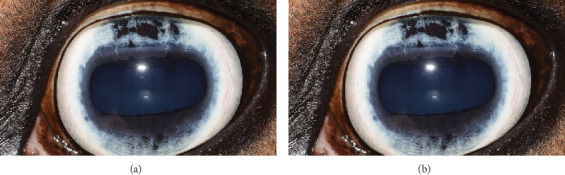
Case 2 at 8-month recheck: (a) OS digital color images and (b) infrared images. Note that the dorsal iris atrophy is visible, with pigment from the deeper iris stroma visible, but with a flat aspect and no recurrence of iris bullae.

An 11-year-old pony mare was referred for ophthalmic evaluation due to an irregularity in the dorsal iris OS. Two dark, protruding, round, cyst-like structures of the dorsal iris were noted by the owner and referring veterinarian. The pony was used for mounted games and exhibited no signs of compromised vision when referred. The ophthalmic examination, PLRs, dazzle reflex, and menace response were all within normal limits OU. The IOP was 28 mmHg OD and 30 mmHg OS (TonoVet). Two dorsal, dark, iridal cyst-like structures, protruding into the anterior chamber without contact with the corneal endothelium (Figure 7), were identified. It was not possible to transilluminate the iris tissue, so a transpalpebral ocular ultrasound (Mindray DP30) examination was performed to confirm iris hypoplasia by ruling out the presence of an iridal mass (e.g., iris melanoma or melanocytoma). Iris hypoplasia was confirmed. Due to the darkly pigmented dorsal iris, diode laser photoablation was proposed to prevent further distension of the iris stroma and possible secondary contact with the corneal endothelium. The pony was sedated with 0.01 mg/kg detomidine, an auriculopalpebral nerve block was performed with 2.5 mL of 2% lidocaine, and topical corneal anesthetic was applied. The two dorsal hypoplastic iris bullae were irradiated with an infrared diode laser (FOX A.R.C. laser) using a laser LIO and a 20D condensing lens. Power output was 1.1 W in continuous wave, and 19 iris points were irradiated for a total of 9.89 J. Complete coagulation and flattening of the anteriorly protruding dorsal iris stroma (hypoplastic tissue) were achieved without any visible secondary complications (Figure 8). Postsurgical therapy was limited to tobramycin–dexamethasone ophthalmic ointment in the OS every 12 h for 5 days and then every 24 h for an additional 5 days, at which time treatments were discontinued. At the 8-month recheck, the previously treated dorsal iris was flat and partially atrophic (Figure 9).

## 3. Discussion

The first case presents a unique and severe form of iris hypoplasia which resulted in anterior synechia of the majority of the dorsal cornea. The resulting changes caused significant visual impairment in this horse's OS and would have likely continued to compound visible changes associated with endothelial dysfunction in the future. Iris hypoplasia is often reported as an incidental finding requiring no additional treatment or intervention. Since obvious clinical variations exist, it is important to contemplate if all of these cases are merely inconsequential incidental findings or if any early treatment options should be pursued prior to significant endothelial contact occurring and anterior synechia developing. Byam-Cook and Knottenbelt reported that a uveal cyst caused recurrent corneal ulceration in a horse due to traumatic endothelial dysfunction and was successfully treated with needle deflation of the cyst and grid keratotomy of the ulceration [7]. Cases receiving intervention after they have developed corneal ulceration are often more difficult to manage and may result in more significant postoperative scarring. And ulceration of the cornea and associated opacification make identification of the underlying cause (i.e., uveal cyst adhered to the corneal endothelium) much more difficult, especially for veterinarians with limited equine ophthalmology experience. Early recognition and treatment may prevent or reduce the risk of visual impairment associated with severe iris hypoplasia. Fortunately, in Case 1, the anterior synechia was still “reducible” despite the chronicity of the lesion. Another question is whether a topical mydriatic prior to referral may have reduced the risk of anterior synechia formation. Long-term mydriatic use may not be ideal given the horse was maintained on pasture and was used as a barrel racer.

Original images sent from the referring veterinarian raised the suspicion of an intraocular or iridal mass due to the severe anterior chamber collapse. Upon presentation to Auburn University, transillumination and retroillumination were sufficient to elicit a tapetal reflex and determine the degree of iris hypoplasia present. While the use of signalment and iris color can help aid in the diagnosis, retroillumination through a thin iris is of utmost diagnostic importance and underscores the importance of adequate lighting, conducting the examination in a dark environment to appropriately visualize the anterior segment changes and to obtain diagnostic quality images.

Alternative treatment options discussed for this horse included attempting pharmacologic dilation of the pupil or performing sector iridectomy to remove the affected tissue. However, despite the pupil dilating following pharmacologic mydriasis, the anterior synechia remained unaffected. The combined use of viscoelastic dissection and mechanical synechiolysis served to accomplish two goals in the present case: first, to separate the iridal tissue from the corneal endothelium to avoid heat dissipation from the iris to the cornea during diode laser ablation and second, to allow for a return of a more natural iris movement that would not obstruct vision. This also allowed for a less invasive procedure to be performed under standing sedation requiring less intense aftercare compared to a sector iridectomy, which would have required a large incision, greater cost, and most likely, general anesthesia. All of which would have also resulted in more intense postoperative care and a greater period of hospitalization. Sector iridectomy may have created a more long-term effect of removing redundant tissue in the first case but may increase the risk of anterior lens luxation given the horse's previously noted vitreal presentation. Sector iridectomy has been described for resection of anterior uveal neoplasia in veterinary patients, such as in a horse with iris melanocytoma, as reported by Scotty et al. This technique was able to salvage the globe and vision but resulted in persistent mydriasis and dyscoria [8]. Potentially, following a sector iridectomy, an iridoplasty technique may have been warranted, as described by Karabaş et al. to reduce the surgically induced mydriasis to facilitate this horse's return to performance and allow it to return to the pasture environment while hopefully minimizing the risks of lens instability [9].

Because a heterochromic iris contains fewer melanocytes and diode laser energy requires pigmented tissue for optimal energy absorption, we were unsure that the diode laser used in this case report would effectively create flow holes via tissue coagulation and contraction. No flow holes were visualized during the procedure but could have easily been obscured by the large amount of redundant iridal tissue present in this case. A more obvious effect during the laser procedure was a contraction of the iridal tissue in response to each laser application at the higher energy settings.

Case 1 developed postoperative ocular hypertension (POH) following the procedure. This may have been due to the viscoelastic alone or a combination of the viscoelastic and intraocular inflammation following the diode laser procedure. There are few reported complications after diode laser deflation of uveal cysts and no documented examples of POH in horses in the literature; however, it has been suggested that cats should be monitored closely for increases in IOP following diode laser ablation [5]. The documented increase in IOP in Case 1 of this case report may have occurred due to increased energy settings causing more significant inflammation. Our energy settings were between 1.5 and 2.0 W and 1500 ms, which were higher than the settings reported by Metzler et al. (400–600 mW and 500 ms). Total energy in those cases used was a maximum of 285 J in comparison to 400 in our Case 1 and 9.89 in Case 2 [5]. Alternatively, viscoelastic obstruction of the iridocorneal angle may have caused the IOP spike, similar to that observed after cataract surgery in dogs and people [10, 11]. The authors of this paper have not identified this as a common phenomenon in horses postoperatively as viscoelastic is routinely left in horses undergoing penetrating keratoplasty or cataract surgery [12]. Treatment of POH in this horse was accomplished with the use of topical steroids with the later addition of a carbonic anhydrase/beta blocker. Aqueocentesis was discussed as a potential option should medical therapy prove to be ineffective. Given the rapid elimination of viscoelastic material from the anterior chamber (observed via direct visualization), it was suspected that this material did not play a substantial role in the POH that occurred in Case 1.

At the 50-day recheck examination, there was a recurrence of the anterior synechia, indicating that the coagulation/contraction of the hypoplastic iris was ineffective at that time point. Despite this, the reduction in the amount of anterior synechia that was achieved resulted in clinical improvement in pupil motility, as evident during the evaluation of the PLRs, and the owner reported an improvement in behavior/vision. The edema, corneal bullae, and POH were transient postoperative effects that improved rapidly following the procedure in Case 1. Given the reoccurrence of synechia, it was recommended that we start a mydriatic agent to reduce the further formation of synechia. At the last recheck (995 days), the mild iris hypoplasia initially present OD had significantly progressed to affect both the dorsal and ventral iris, causing severe miosis. Progression of the findings OS had increased mildly, leading to recurrence of anterior synechia, but with a functional pupil. This case report indicates that iris hypoplasia can be a progressive condition and, in certain cases, may contribute to vision impairment.

Case 2 was less progressive upon presentation, and diode laser ablation was more effective, leading to more pronounced results with longer lasting effects. The surgical procedure was also less invasive, with no observed complications. This suggests that earlier recognition of iris hypoplasia and intervention may result in more effective long-term results in select cases.

## 4. Conclusions

Diode laser provides an alternative treatment option for iris hypoplasia with a cyst-like presentation. However, earlier intervention (ideally before anterior synechia formation occurs) may be the key in obtaining a more desirable treatment outcome with a less invasive technique not requiring intraocular manipulation.

## Data Availability

Data sharing is not applicable to this article as no datasets were generated or analyzed during the current study. All pertinent case information remains part of the medical records at Auburn University (20-year-old Quarter Horse) and Cascina Gufa Equine Clinic (11-year-old pony mare). The initial working case report draft and additional images can be found with the authors.
